# Symbiont-Induced Phagosome Changes Rather than Extracellular Discrimination Contribute to the Formation of Social Amoeba Farming Symbiosis

**DOI:** 10.1128/spectrum.01727-21

**Published:** 2022-04-20

**Authors:** Yuehui Tian, Tao Peng, Zhenzhen He, Luting Wang, Xurui Zhang, Zhili He, Longfei Shu

**Affiliations:** a Environmental Microbiomics Research Center, School of Environmental Science and Engineering, Southern Marine Science and Engineering Guangdong Laboratory (Zhuhai), Sun Yat-sen University, Guangzhou, China; University of Vienna

**Keywords:** symbiosis, chemotaxis, phagosome, discrimination, *Dictyostelium discoideum*, *Burkholderia*

## Abstract

Symbiont recognition is essential in many symbiotic relationships, especially for horizontally transferred symbionts. Therefore, how to find the right partner is a crucial challenge in these symbiotic relationships. Previous studies have demonstrated that both animals and plants have evolved various mechanisms to recognize their symbionts. However, studies about the mechanistic basis of establishing protist-bacterium symbioses are scarce. This study investigated this question using a social amoeba Dictyostelium discoideum and their *Burkholderia* symbionts. We found no evidence that D. discoideum hosts could distinguish different *Burkholderia* extracellularly in chemotaxis assays. Instead, symbiont-induced phagosome biogenesis contributed to the formation of social amoeba symbiosis, and D. discoideum hosts have a higher phagosome pH when carrying symbiotic *Burkholderia* than nonsymbiotic *Burkholderia*. In conclusion, the establishment of social amoeba symbiosis is not linked with extracellular discrimination but related to symbiont-induced phagosome biogenesis, which provides new insights into the mechanisms of endosymbiosis formation between protists and their symbionts.

**IMPORTANCE** Protists are single-celled, extremely diverse eukaryotic microbes. Like animals and plants, they live with bacterial symbionts and have complex relationships. In protist-bacterium symbiosis, while some symbionts are strictly vertically transmitted, others need to reestablish and acquire symbionts from the environment frequently. However, the mechanistic basis of establishing protist-bacterium symbioses is mostly unclear. This study uses a novel amoeba-symbiont system to show that the establishment of this symbiosis is not linked with extracellular discrimination. Instead, symbiont-induced phagosome biogenesis contributes to the formation of social amoeba-bacterium symbiosis. This study increases our understanding of the mechanistic basis of establishing protist-bacterium symbioses.

## INTRODUCTION

Host-symbiont mutualisms are prevalent in nature and can significantly impact each other’s fitness (1–5). While some symbionts are vertically transmitted, other symbiotic relationships need to reestablish in every generation and acquire symbionts from the environment (6). Therefore, how to find the right partner is a crucial challenge in their relationship. It has been demonstrated that both animals and plants, such as the legume and squid symbioses, have evolved various mechanisms to recognize their symbionts (7, 8). However, it is unclear whether unicellular protist hosts could recognize and discriminate their symbionts.

Protists are unicellular eukaryotic organisms that are not animals, plants, or fungi, which have complex relationships with bacteria, ranging from predation to symbiosis (1, 9–11). For instance, a large number of diverse symbionts can be found in both ciliates (12–17) and amoebas (1, 18). Because protists are difficult to culture, and most of their symbionts are unculturable bacteria, our understanding is restricted to a few systems (1, 13, 19–24), and we know very little about the partner choice in protist-bacteria interactions (25). Therefore, we need simple systems in which both partners can be manipulated empirically.

The amoeba proto-farming symbiosis is a promising system to address whether unicellular protist hosts could recognize and discriminate their symbionts (19–21, 25–28). Dictyostelium discoideum is a soil-dwelling amoeba belonging to protozoa and primarily feeding on bacteria, which has been widely used as an ideal system to study cell biology, symbiosis, evolution, and ecology (20, 21, 23, 24, 26, 28–32). Amoebas can aggregate and differentiate into pluricellular fruiting bodies upon food-deprived conditions. Approximately 20% of cells sacrifice to generate stalk, and the remaining cells differentiate into mature spores, resulting in a sorus at the top of the stalk (21). *Burkholderia agricolaris* and *B. hayleyella*, two symbiotic bacteria, can form a stable association with D. discoideum hosts. They could not support amoeba growth alone, but they benefit the amoebas by inducing additional bacterial carriage, which can be used to seed new food populations (20, 21). Both symbionts can live on their own, indicating they are facultative symbionts, which raises the question of how the association between D. discoideum and its carried *Burkholderia* is formed and maintained.

Dictyostelium discoideum is a prime organism to study host-bacterium interactions (33). Our previous study showed that *Burkholderia* symbionts used chemotaxis to find their amoeba hosts (25). However, it is unclear whether D. discoideum hosts could recognize and discriminate their *Burkholderia* symbionts. Amoebas interact with bacteria through two steps. First, they use chemotaxis to search and track bacteria. Chemotaxis is the movement of cells toward a chemical gradient, which has significant roles in many biological processes (34). It has been reported that amoebas are attracted to Gram-negative bacteria in a chemotaxis assay (35), but it is not clear whether D. discoideum hosts are more attracted to their *Burkholderia* symbionts. Second, amoebas use phagocytosis to ingest and feed on bacteria as phagocytes. After engulfment, the phagosomes of amoebas play essential roles in killing and digesting bacteria with the help of acidification, proteases, hydrolases, and ROS (36–39). Previous studies in other systems have shown that the evasion of the lysosomal fusion of the phagosome is mainly due to bacterial mechanisms. It was reported that some components such as ankyrin proteins and MavE effector of L. pneumophila have effects on the interaction with hosts via phagosome biogenesis and lysosomal evasion (40, 41). Bacterial surface traits, including alkaline substances, can partially inhibit the digestion of Tetrahymena pyriformis from enhancing escape rates (42). Some pathogenic bacteria can often survive from phagosome acidification and exist in amoebas by inhibiting phagosome maturation or escaping from phagosomes (18, 43), whereas most bacteria cannot survive within amoebas. Therefore, the bacterium-induced phagosome changes may also contribute to the formation of social amoeba farming symbiosis.

Currently, it is unclear why some bacteria can form symbiotic relationships with D. discoideum hosts while others cannot. In addition, the debate also exists whether hosts can discern symbiotic, nonsymbiotic bacteria, or food bacteria to stabilize relationships with symbiotic bacteria. It may not be accidental that hosts can selectively discriminate bacteria, forming symbiotic relationships in the environment. Therefore, we hypothesize that D. discoideum hosts can distinguish and recognize their *Burkholderia* symbionts extracellularly (chemotaxis) and intracellularly (phagocytosis). We conducted chemotaxis assays and flow cytometry measurements on phagosome pH to answer the following question: can amoeba hosts discriminate their bacterial symbionts extracellularly or intracellularly?

## RESULTS

### *D. discoideum* moved to all bacteria in a one-way chemotaxis assay, but the chemotactic responses were similar.

**FIG 1:**
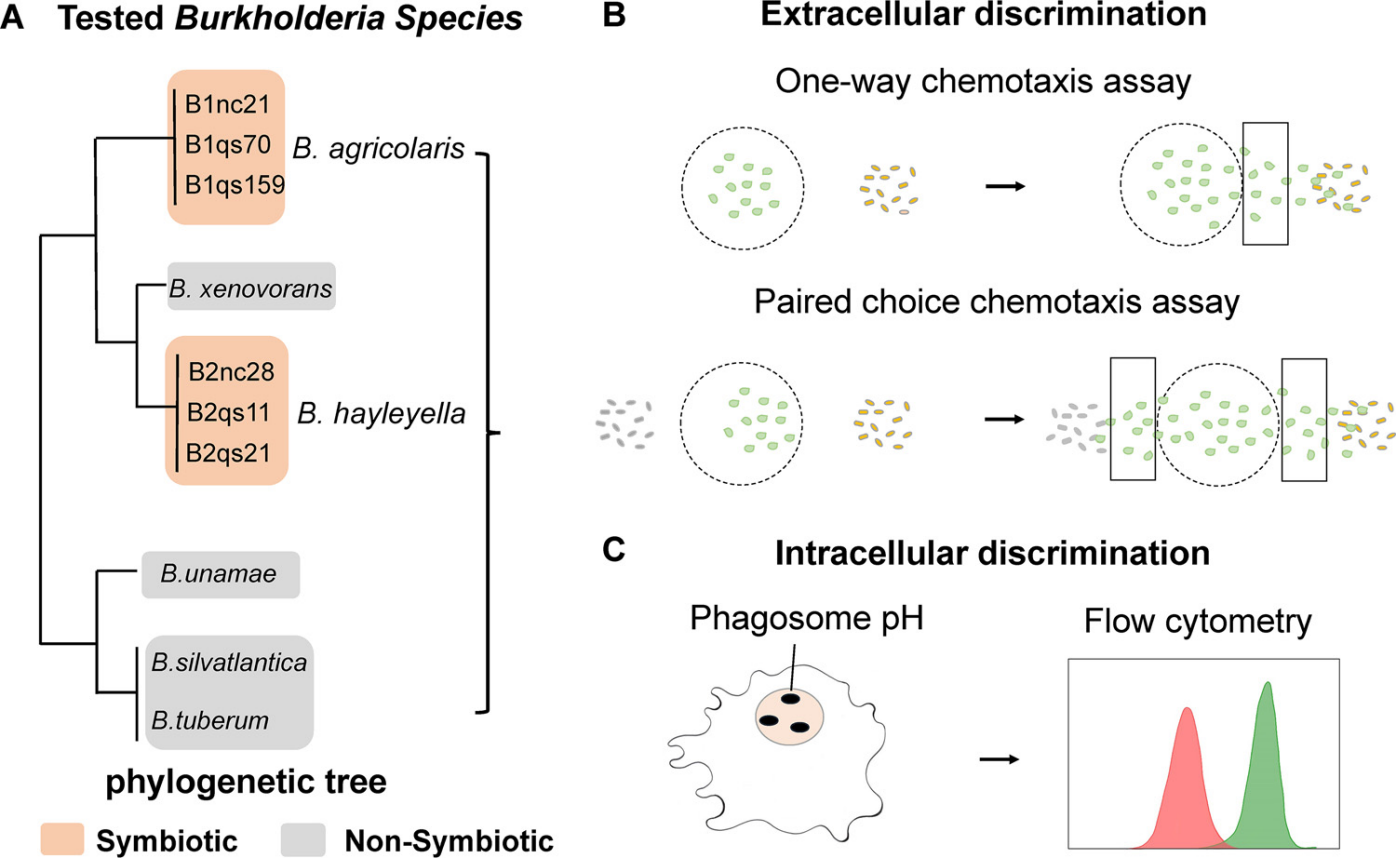
Flow chart of the experimental design. (A) K. pneumoniae and 10 *Burkholderia* isolates, including 6 carried *Burkholderia* (in orange boxes) and 4 noncarried *Burkholderia* (in gray boxes) described previously (20, 50), were used. (B) Amoeba cells are depicted in green in the round circles. In the extracellular discrimination, these bacteria were tested using one-way chemotaxis and a paired-choice assay, respectively, and the number of migrated amoebas toward bacteria was counted through the black boxes. (C) In the intracellular discrimination, phagosome pH was measured using the pH-sensitive probe on flow cytometry.

**FIG 2:**
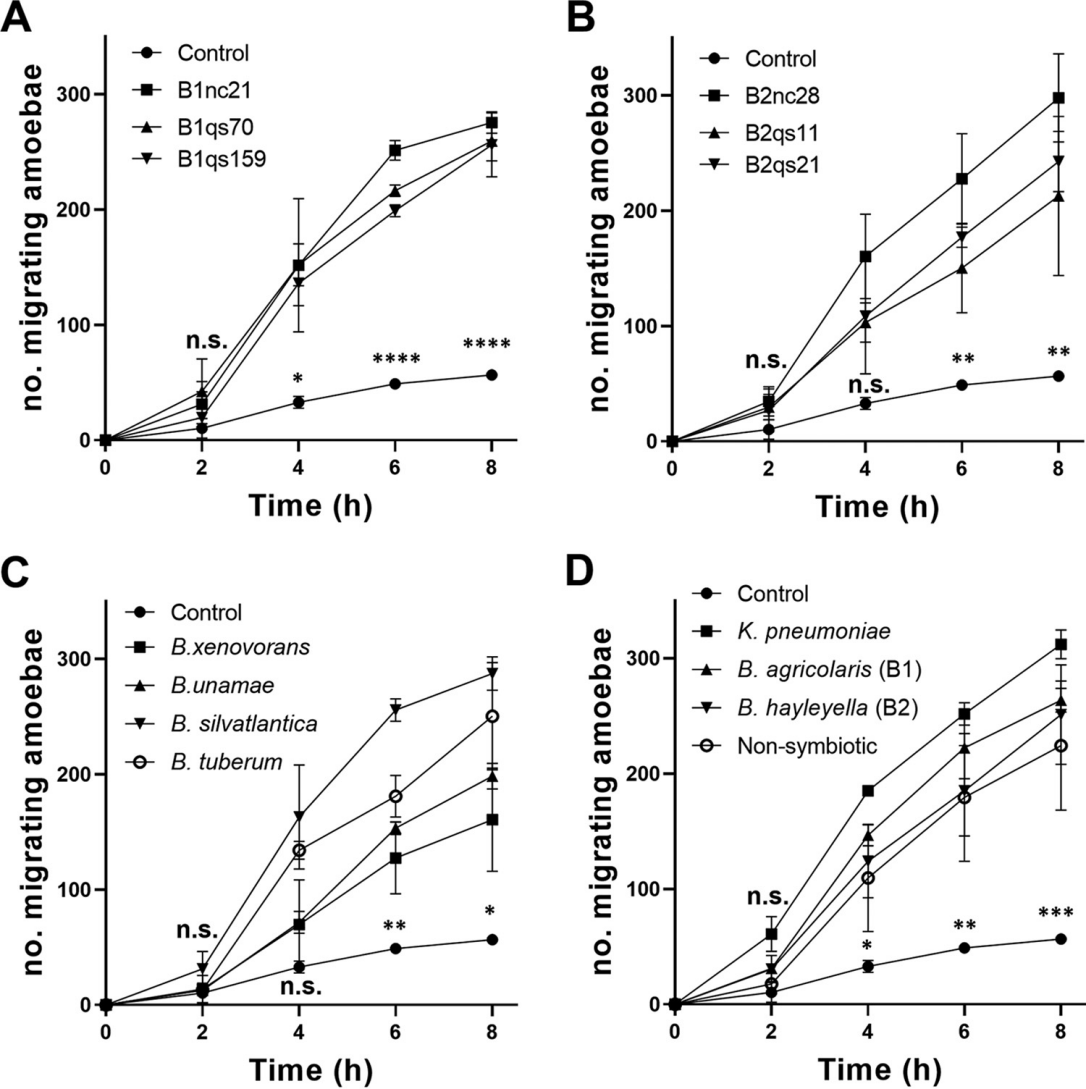
One-way chemotaxis assay of D. discoideum amoebas toward different bacteria. (A to C) Amoeba migration in the presence of *B. agricolaris* (B1nc21, B1qs70, and B1qs159), *B. hayleyella* (B2nc28, B2qs11, and B2qs21), and four nonsymbiotic bacteria (*B. xenovorans*, B. unamae, *B. silvatlantica*, and *B. tuberum*) compared to the control, respectively. (D) Amoebas show chemotaxis responses to *K. pneumoniae*, *B. agricolaris*, *B. hayleyella*, and nonsymbiotic bacteria at 2, 4, 6, and 8 h, respectively (*n* = 3; all error bars represent the SD). Statistical analyses were performed using one-way ANOVA and Tukey’s multiple-comparison test. Significant differences between data points of the control group and data points of the lowest testing group were labeled out (*, *P < *0.05; **, *P < *0.01; ***, *P < *0.001; ****, *P < *0.0001; n.s., not significant).

We conducted one-way chemotaxis assays to investigate how D. discoideum host responded to food source bacteria K. pneumoniae, symbiotic and nonsymbiotic *Burkholderia* species (Fig. 1). The numbers of migrated amoebas gradually increased in all treatments with time (2, 4, 6, and 8 h; Fig. 2). By one-way analysis of variance (ANOVA) using Tukey’s multiple-comparison test, no significant difference in migrating amoebas responding to all the bacteria was observed at the beginning of 2 h. However, compared to the control group, D. discoideum showed stronger chemotaxis to all targeted bacteria than blank control after 6 h (Fig. 2A to C).

Within carried *Burkholderia* symbionts, our results showed that their chemotactic responses were similar, and no significant variation was observed within each symbiotic *Burkholderia* species at 8 h (*P* > 0.05) (Fig. 2A and B). However, nonsymbiotic *Burkholderia* species induced different chemotactic responses, in which fewer amoebas were attracted to *B. xenovorans* than to other *B. silvatlantica* (*P* < 0.0001) and *B. tuberum* (*P* = 0.02) after 6 h (Fig. 2C).

Overall, amoebas showed positive chemotactic responses to symbiotic *B. agricolaris* (*P* = 0.0007), *B. hayleyella* (*P* = 0.0048), non-symbiotic *Burkholderia* (*P* = 0.004), and the food source K. pneumoniae (*P* = 0.0002) (Fig. 2D). However, there was no significant difference in amoebas migrating toward the symbiotic *B. agricolaris* and *B. hayleyella* compared to nonsymbiotic species (*P* > 0.05) (Fig. 2D).

### *D. discoideum* could not discriminate different bacteria in a paired choice assay.

**FIG 3:**
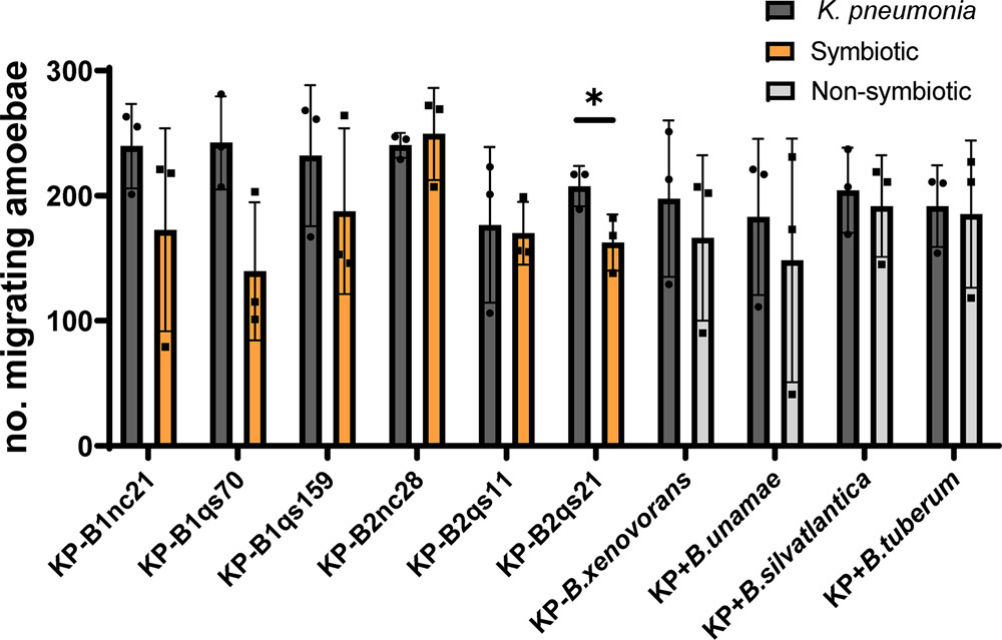
Paired-choice assay between K. pneumoniae and each *Burkholderia* species. Each bar chart showed paired choice assay between K. pneumoniae and individual *Burkholderia* species. The dark bars indicate the migrating number of K. pneumoniae compared to each symbiotic (orange bars) or nonsymbiotic *Burkholderia* (gray bars). No significance was detected between K. pneumoniae and most individual bacteria by using an unpaired *t* test (*n* = 3; all error bars represent the SD). The statistical analysis was performed using an unpaired *t* test. We predicted that amoebas preferred a symbiotic strain to K. pneumoniae. However, the results showed that amoebas could not distinguish them and, in one case, even preferred K. pneumoniae, which rejected our hypothesis.

Furthermore, we performed paired choice assays to investigate how D. discoideum host responded to different bacteria. We separately compared food bacterium *K. pneumonia* with the other 10 individual bacteria in a paired choice assay and analyzed with a two-tailed Student *t* test. Our data showed that the number of migrated cells toward bacteria is similar between feeding bacteria and each symbiotic or nonsymbiotic *Burkholderia* species, suggesting that amoebas show no significant difference of chemotaxis between *Klebsiella pneumoniae* and most bacterial species (*P* > 0.05) except for B2qs21 (*P* = 0.0481) (Fig. 3).

**FIG 4:**
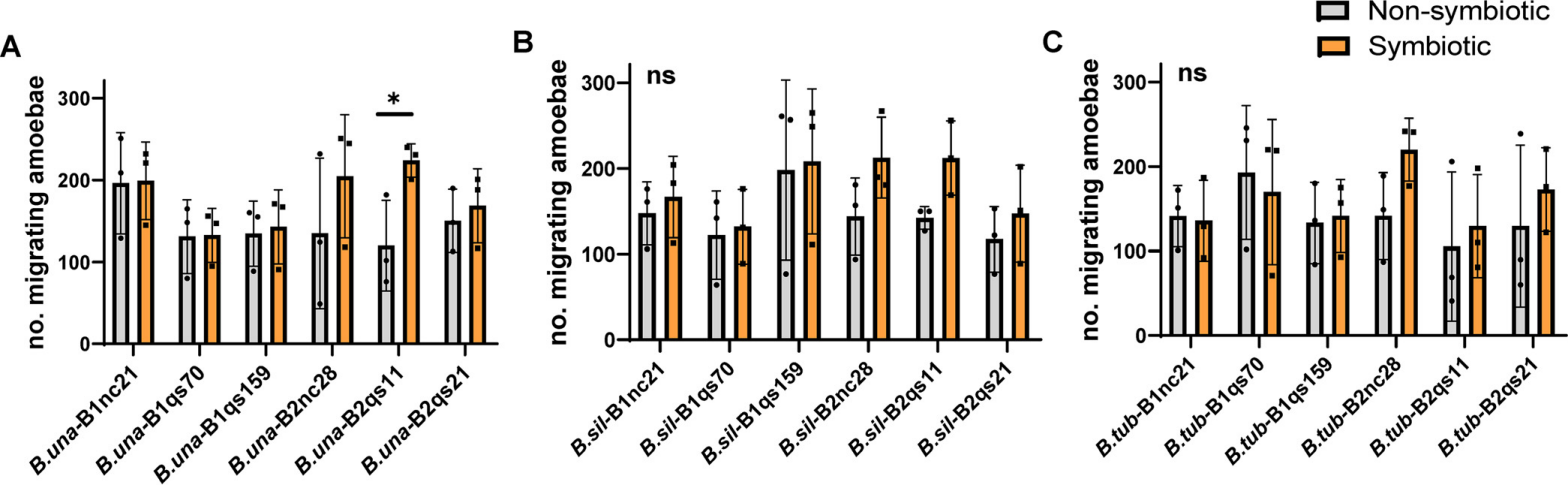
Paired-choice assay between symbiotic and nonsymbiotic *Burkholderia* species. Each nonsymbiotic bacterium—B. unamae (A), *B. silvatlantica* (B), and *B. tuberum* (C)—was separately compared to individual symbiotic *Burkholderia* samples. The abbreviated names *B.una*, *B.sil*, and *B.tub* are used in panels A to C. The gray bars indicate the migrating numbers of amoebas when comparing each nonsymbiotic with other individual symbiotic *Burkholderia* (orange bars). Only clone *B. unamae* paired against B2qs11 shows a significant difference for migrating amoebas (*, *P* < 0.05; *n* = 3; the error bar represents the SD). The statistical analysis was performed using an unpaired *t* test.

We next compared each nonsymbiotic *Burkholderia* (including B. unamae, *B. silvatlantica*, and *B. tuberum*) to the other six symbiotic *Burkholderia* for the paired-choice assay. The results showed that only one combination (B. unamae versus *B. agricolaris* B2qs11) had a significant difference in *Dictyostelium* migration (*P* = 0.0377) (Fig. 4), while no significant difference was observed in all other comparisons. These results suggest that D. discoideum host cannot discriminate symbiotic and nonsymbiotic *Burkholderia* species extracellularly.

### *Burkholderia* bacteria change the phagosome pH of *D. discoideum* host.

**FIG 5:**
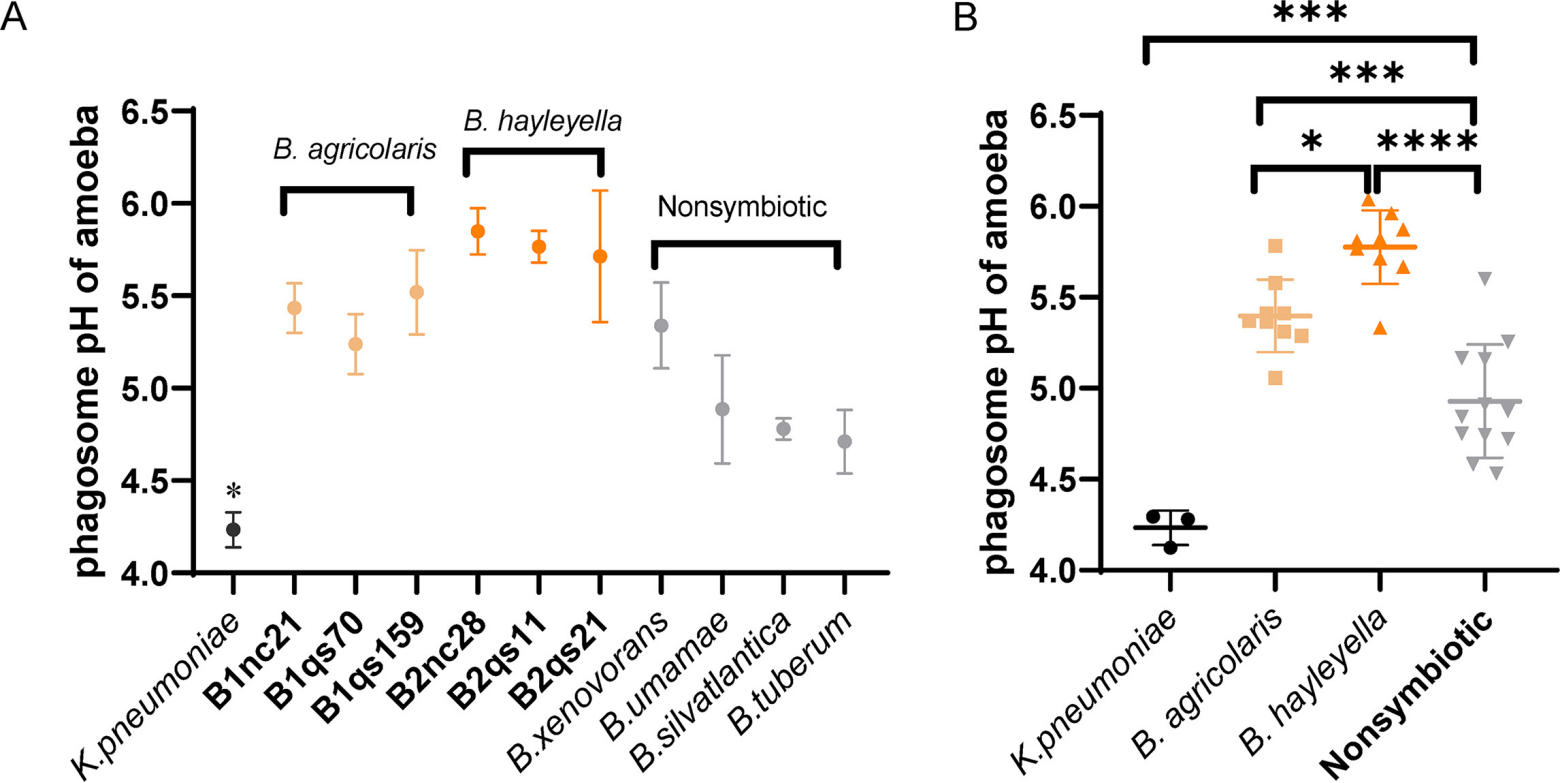
Phagosome pH of amoebas in response to different bacteria. The same experiment was plotted separately by bacterium (A) and group (B). (A) Comparison among different strains. Compared to other different clones, the pH was changed significantly in amoebas with *K. pneumonia* (*n* = 3; the error bar represents the SD). (B) Comparison among symbiotic and nonsymbiotic bacteria. For panels A and B, the dark symbols indicate the phagosome pH of amoebas responding to *K. pneumonia*. The light orange and the deep orange symbols indicate the phagosome pH of amoebas responding to symbiotic *B. agricolaris* and *B. hayleyella*, respectively. The gray symbols indicate the phagosome pH of amoebas responding to the nonsymbiotic bacteria B. unamae, *B. silvatlantica*, and *B. tuberum.* For panels A and B, asterisks indicate significance (*, *P < *0.05; ***, *P < *0.001; ****, *P < *0.0001) according to the one-way ANOVA Tukey’s multiple-comparison test.

Since we found that D. discoideum cannot discriminate its symbionts bacteria extracellularly, next, we investigated whether it could discriminate them intracellularly. Using pH-sensitive fluorescent probes, we measured the phagosome pH by flow cytometry (see Table S1 in the supplemental material). By one-way ANOVA Tukey’s multiple-comparison test, we observed that the phagosome pH of D. discoideum cells infected with *Burkholderia* was significantly different compared to food bacterium K. pneumoniae except for *B. silvatlantica* and *B. tuberum* (*P* > 0.05) (Fig. 5A).

In addition, nonsymbiotic *Burkholderia* induced the lowest phagosome pH compared to symbiotic *B. agricolaris* (*P* = 0.0008) and *B. hayleyella* (*P* < 0.0001) (Fig. 5B). Furthermore, we also observed differences between two symbiotic *Burkholderia*: *B. hayleyella* induced a higher phagosome pH than did *B. agricolaris* (*P* = 0.0132) (Fig. 5B). These results indicated that symbiotic *Burkholderia* could inhibit phagosome acidification of D. discoideum host.

## DISCUSSION

The symbiotic associations between protists and their symbionts provide an excellent system to study symbiosis because we could culture, mix, and match both partners to test different research questions in ecology and evolution (1, 22). Using D. discoideum as a host system, this study showed that symbiont-induced phagosome changes rather than extracellular discrimination contributed to the formation of social amoeba farming symbiosis. We found no evidence that D. discoideum could distinguish different *Burkholderia* extracellularly in chemotaxis assays. Instead, symbiont-induced phagosome biogenesis contributed to the formation of social amoeba symbiosis, and D. discoideum hosts exhibited higher phagosome pH when carrying symbiotic *Burkholderia* than nonsymbiotic *Burkholderia*.

We found no evidence that amoeba hosts could recognize their symbionts extracellularly. Previous studies have shown that amoebas locate and search prey effectively depending on chemotaxis. It was reported that soluble compounds of secondary metabolites produced by bacteria mediate interactions between *Dictyostelium* and bacteria (44, 45). Consequently, D. discoideum has an instinctive response to feed on bacteria or acquire symbionts from the complex environment. Recent studies indicate an intense preference for Gram-negative compared to Gram-positive bacteria (30). However, our study showed that all tested *Burkholderia* bacteria could attract amoebas, but the cells could not distinguish symbiotic and nonsymbiotic *Burkholderia* bacteria. We also found that D. discoideum cannot distinguish K. pneumoniae paired against other *Burkholderia*, although D. discoideum showed a more robust response to K. pneumoniae in some cases.

Our results support the hypothesis that amoebas may not discriminate different *Burkholderia* species extracellularly. In addition, the results show that symbiont-induced phagosome biogenesis contributes to the formation of social amoeba symbiosis. Bacteria have complex relationships with amoebas, evolving complex intracellular lifestyles (1). For example, amoebas play roles such as bacterial predators, symbiotic partners, bacterial vehicles or “Trojan horses” and “biological reservoirs” (46). Recent studies have reported that some microorganisms can resist killing by free-living amoebas (47). Some pathogenic bacteria can resist digestion and escape from amoebas to avoid damage from the phagosome, reproduce within the environment, and exploit host resources (48). Our results suggested that specific mechanisms of the symbiotic *Burkholderia* are to change the pH of the phagosome. As a result, intracellular pathogens would affect phagosome-lysosome fusion (48), which may explain how the symbiotic *Burkholderia* can survive in the phagosome and form a stable symbiotic relationship with the host *Dictyostelium*. Interestingly, although the nonsymbiotic *Burkholderia* species have similar edibility to the amoeba host (49), one species *B. xenovorans*, induced a higher phagosome pH than B. unamae, *B. silvatlantica*, and *B. tuberum*. In addition, phylogenetically, *B. xenovorans* is also closer to the symbiotic *Burkholderia*, indicating a possible correlation between phylogeny and phagosome acidification disruption.

This study also provides new insights into the relationships between *Dictyostelium* and *Burkholderia* and suggests a potential mechanism of bacterial food carrying. Symbiotic *Burkholderia* bacteria have a similar survival strategy with pathogens, and previous studies also showed that harboring *Burkholderia* imposed fitness costs on *Dictyostelium* hosts (19, 20). Therefore, we believe that the *Dictyostelium*-*Burkholderia* symbiosis is or has evolved from a more parasitic relationship. In addition, the induction of bacterial food carrying is likely the result of symbiont-induced phagosome changes. Only symbiotic *Burkholderia* can form a stable relationship with amoeba and induced bacterial carriage instead of nonsymbiotic *Burkholderia*. This symbiotic specificity mechanism occurs due to pH variations in the intracellular environment. Therefore, a higher phagosome pH plays a crucial role in maintaining *Dictyostelium*-*Burkholderia* symbiosis, which in turn creates a moderate host niche that allows other food bacteria to survive. Future research should focus on the precise molecular mechanisms of the inhibition of phagosome acidification in these symbiotic *Burkholderia* bacteria.

## MATERIALS AND METHODS

### *Dictyostelium* strains and culture conditions.

Wild D. discoideum clones QS9 was used in this study (21). Frozen D. discoideum spores were grown on SM/5 agar plates (2 g glucose, 2 g Bacto peptone [Oxoid], 2 g yeast extract [Oxoid], 0.2 g MgCl_2_, 1.9 g KH_2_PO_4_, 1 g K_2_HPO_4_, and 15 g agar per L), mixed with the food bacterium K. pneumoniae, and cultured in a light incubator at 21°C.

### Bacterial strains and culture conditions.

K. pneumoniae and 10 *Burkholderia* isolates, including 6 carried *Burkholderia* and 4 noncarried *Burkholderia* described in previous work (20, 50), were used in this study. The symbiotic *Burkholderia* contains *B. agricolaris* (B1qs70, B1qs159, and B1nc21) and *B. hayleyella* (B2qs11, B2qs21, and B2nc28). The bacteria from the frozen clonal isolate were incubated on SM/5 agar medium for approximately 2 to 5 days at 21°C. Bacterial strain information is presented in Table 1.

**TABLE 1:** Bacterial strains used in this study

Bacterial strain	Type	Gram stain	Reference
*B. agricolaris*			
B1qs70	Symbiotic	Gram negative	21
B1qs159	Symbiotic	Gram negative	21
B1nc21	Symbiotic	Gram negative	21
*B. hayleyella*			
B2qs11	Symbiotic	Gram negative	21
B2qs21	Symbiotic	Gram negative	21
B2nc28	Symbiotic	Gram negative	21
B. unamae	Nonsymbiotic	Gram negative	25
*B. tuberum*	Nonsymbiotic	Gram negative	25
*B. silvatlantica*	Nonsymbiotic	Gram negative	25
*B. xenovorans*	Nonsymbiotic	Gram negative	25
K. pneumoniae	Nonsymbiotic	Gram negative	21

### Extracellular discrimination: chemotaxis assay.

Based on the 16S rRNA gene phylogeny (20, 25), two distinct clades, including symbiotic *B. agricolaris* and *B. hayleyella*, were selected for chemotaxis assays together with four nonsymbiotic *Burkholderia* bacteria (Fig. 1). To detect the diverse chemotactic responses of D. discoideum to different bacteria, the methods were shown as follows. 2 × 10^5^ spores were suspended with 200 μL of K. pneumoniae (optical density at 600 nm [OD_600_] = 1.5) in starvation buffer (2.2 g KH_2_PO_4_ and 0.7 g K_2_HPO_4_ per L). Amoeba log growth occurs about 36 h after plating spores (28). At this time, log-growth amoebas were collected in the starvation buffer from the petri dishes for the chemotaxis experiment and centrifuged the collected amoebas/bacterial suspension at 1,500 × *g* for 3 min to wash the amoebas clean from the bacteria.

The pelleted amoebas were washed in an excess volume of ice-cold starvation buffer three or four times to get rid of residual bacteria. Each bacterial suspension in starvation buffer was prepared at an OD_600_ of ~1.5. Furthermore, we tested one-way chemotaxis and paired-choice assay, respectively, 2 μL of amoeba suspension was spotted on 2% Noble agar, and 2 μL of each bacterial suspension was spotted onto one side or two different bacterial pair on both sides, which was measured at a 0.65-cm distance from the amoeba suspension. A grid was placed beneath the plate to ensure equal distances. After being spotted at room temperature, the number of migrated amoebas that moved toward bacteria was counted at different time points (2, 4, 6, and 8 h). We used a microscope with 20× lens objective (200× total magnification) in the bright field to manually count the migrated amoeba numbers (Fig. 1B). All tests were done in three biological replicates.

### Measurement of phagosome pH using flow cytometry.

Log-growth amoebas infected with different *Burkholderia* samples were used in this study. To set up the experiment, we mixed the specified *Burkholderia* (OD_600_ = 1.5) at 5% (10 μL) and K. pneumoniae at a 95% (190 μL) volume and plated D. discoideum spores (2 × 10^5^) with 200 μL of the bacterial mixture on SM/5 plates in a light incubator at 21°C. The amoeba cells at the exponential stage were collected after 36 h. The amoeba suspension was collected and rinsed three times at 1,500 × *g* for 3 min to remove the remaining bacteria. Cells were incubated with dextran coupled to Oregon green (250 μg/mL; Invitrogen), a pH-sensitive probe combined, and a pH-insensitive probe Alexa 647 (30 μg/mL; Invitrogen) to label amoebas that carried symbiotic *Burkholderia* or nonsymbiotic *Burkholderia* (51). After 20 min, the cells were pelleted (1,500 × *g*, 3 min) and rinsed once for further flow cytometer analyses. All tests were done in three biological replicates.

A flow cytometer (Accuri C6 cytometer; BD, USA) was used to measure endosomal pH in cells. The FL1 channel was applied to measure the Oregon green fluorescence with an excitation wavelength at 488 nm and an emission of 515 to 545 nm, while the FL4 channel was used to measure Alexa 647 fluorescence with an excitation of 632 nm and an emission of 655 to 695 nm. At least 10,000 cells were detected, with the median fluorescence values (see Table S1 in the supplemental material). The background autofluorescence values were subtracted from cells without exposure to fluorescent dyes. A calibration curve was prepared in each experiment. After being mixed with fluorescent dextran for 20 min, the cells were washed and resuspended in ice-cold HL5 at the indicated pH values (pH 3, 4, 5, 6, 7, and 8) and supplemented with 0.1% (wt/vol) sodium azide and 40 mM NH_4_Cl before fluorescence-activated cell sorting analysis, and calibration curves were determined (51).

### Statistical analyses.

All statistical analyses were performed by using the GraphPad Prism 8 software package. The results are shown as means ± the standard deviations (SD) in the figure legends. In Fig. 2 and 5, statistical analyses were performed using ANOVA with Tukey’s multiple-comparison test. In Fig. 3 and 4, data were analyzed using an unpaired two-tailed Student *t* test.
