# Supplementary material for: Symbiont-Induced Phagosome Changes Rather than Extracellular Discrimination Contribute to the Formation of Social Amoeba Farming Symbiosis
Source: Microbiol Spectr. 2022 Apr 20;10(3):e01727-21. doi: 10.1128/spectrum.01727-21 (PMC9241765; doi:10.1128/spectrum.01727-21)
Supplement: SUPPLEMENTAL FILE 1 — Supplemental material. Download spectrum.01727-21-s001.pdf, PDF file, 0.1 MB [file spectrum.01727-21-s001.pdf]

Table S1. Details of the measurement of amoeba phagosome pH in the flow cytometer experiment.

Group	Samples	Replicate	Count	Alexa 647	Oregon Green	Ratio	pH
Kp	Kp	3	51290	51958	10067	0.19	4.12
Kp	Kp	1	56596	53138	11305	0.21	4.28
Kp	Kp	2	53177	53221	11412	0.21	4.29
Symbiotic	B1nc21	3	49068	12310	4845	0.39	5.31
Symbiotic	B1nc21	2	56225	11465	4793	0.42	5.41
Symbiotic	B1nc21	1	57352	17499	8076	0.46	5.58
Symbiotic	B1qs70	1	59206	19459	6582	0.34	5.06
Symbiotic	B1qs70	2	63536	13479	5234	0.39	5.29
Symbiotic	B1qs70	3	46459	14837	6051	0.41	5.37
Symbiotic	B1qs159	3	26952	19935	8106	0.41	5.37
Symbiotic	B1qs159	1	31794	18265	7632	0.42	5.41
Symbiotic	B1qs159	2	28035	18582	9687	0.52	5.78
Symbiotic	B2nc28	3	221914	5172	2590	0.50	5.71
Symbiotic	B2nc28	2	231581	5345	2941	0.55	5.87
Symbiotic	B2nc28	1	258692	7272	4220	0.58	5.96
Symbiotic	B2qs11	3	202472	5105	2485	0.49	5.67
Symbiotic	B2qs11	1	97910	6024	3191	0.53	5.81
Symbiotic	B2qs11	2	126570	9274	4950	0.53	5.82
Symbiotic	B2qs21	3	12349	19341	7713	0.40	5.33
Symbiotic	B2qs21	1	15719	22967	11877	0.52	5.77
Symbiotic	B2qs21	2	14773	22685	13793	0.61	6.04
Nonsymbiotic	*B. xenovorans*	1	32634	23323	8386	0.36	5.16
Nonsymbiotic	*B. xenovorans*	2	24618	21416	8152	0.38	5.26
Nonsymbiotic	*B. xenovorans*	3	23407	20832	9751	0.47	5.60
Nonsymbiotic	*B. umamae*	3	25951	27917	7106	0.25	4.58
Nonsymbiotic	*B. umamae*	1	27919	25971	8049	0.31	4.91
Nonsymbiotic	*B. umamae*	2	27917	26217	9459	0.36	5.17
Nonsymbiotic	*B.silvatlantica*	1	194631	11329	3175	0.28	4.74
Nonsymbiotic	*B.silvatlantica*	3	167640	8553	2409	0.28	4.75
Nonsymbiotic	*B.silvatlantica*	2	178715	8563	2552	0.30	4.85
Nonsymbiotic	*B. tuberum*	2	132215	11422	2825	0.25	4.53
Nonsymbiotic	*B. tuberum*	3	117653	9767	2705	0.28	4.72
Nonsymbiotic	*B. tuberum*	1	124913	20912	6347	0.30	4.88
